# Fussy Feeders: Phyllosoma Larvae of the Western Rocklobster (*Panulirus cygnus*) Demonstrate Prey Preference

**DOI:** 10.1371/journal.pone.0036580

**Published:** 2012-05-07

**Authors:** Megan I. Saunders, Peter A. Thompson, Andrew G. Jeffs, Christin Säwström, Nikolas Sachlikidis, Lynnath E. Beckley, Anya M. Waite

**Affiliations:** 1 Oceans Institute & School of Environmental Systems Engineering, University of Western Australia, Crawley, Western Australia, Australia; 2 Global Change Institute, University of Queensland, St. Lucia, Queensland, Australia; 3 Australian Commonwealth Scientific Industrial and Research Organisation, Hobart, Tasmania, Australia; 4 Leigh Marine Laboratory, University of Auckland, Warkworth, Northland, New Zealand; 5 Department of Primary Industries and Fisheries, Queensland Government, Cairns, Queensland, Australia; 6 School of Environmental Science, Murdoch University, Murdoch, Western Australia, Australia; University of Western Ontario, Canada

## Abstract

The Western Rocklobster (*Panulirus cygnus*) is the most valuable single species fishery in Australia and the largest single country spiny lobster fishery in the world. In recent years a well-known relationship between oceanographic conditions and lobster recruitment has become uncoupled, with significantly lower recruitment than expected, generating interest in the factors influencing survival and development of the planktonic larval stages. The nutritional requirements and wild prey of the planktotrophic larval stage (phyllosoma) of *P. cygnus* were previously unknown, hampering both management and aquaculture efforts for this species. Ship-board feeding trials of wild-caught mid-late stage *P. cygnus* phyllosoma in the eastern Indian Ocean, off the coast of Western Australia, were conducted in July 2010 and August-September 2011. In a series of experiments, phyllosoma were fed single and mixed species diets of relatively abundant potential prey items (chaetognaths, salps, and krill). Chaetognaths were consumed in 2–8 times higher numbers than the other prey, and the rate of consumption of chaetognaths increased with increasing concentration of prey. The highly variable lipid content of the phyllosoma, and the fatty acid profiles of the phyllosoma and chaetognaths, indicated they were from an oligotrophic oceanic food chain where food resources for macrozooplankton were likely to be constrained. Phyllosoma fed chaetognaths over 6 days showed significant changes in some fatty acids and tended to accumulate lipid, indicating an improvement in overall nutritional condition. The discovery of a preferred prey for *P. cygnus* will provide a basis for future oceanographic, management and aquaculture research for this economically and ecologically valuable species.

## Introduction

Effects of environmental variability on fisheries are of primary concern to management of these important resources. For meroplanktonic species, identifying oceanographic processes influencing larvae during their planktonic period is of critical importance to understanding the recruitment processes that could underpin fluctuations in the adult population [1]. Due to increasing pressures on marine resources worldwide, unraveling environmental mechanisms influencing recruitment of commercially important species is essential to fisheries management.

Spiny lobsters (Decapoda, Palinuridae) form the basis of some of the most commercially valuable fisheries worldwide [2]. The Western Rocklobster (*Panulirus cygnus*) is Australia's largest single species fishery, valued at AUD $200 million a year [3], [4]. The annual commercial catch has varied between 5,500 and 14,500 t over the last 30 years largely due to fluctuations in recruitment [4]. In 2000, and again in 2006, the fishery was certified by the international Marine Stewardship Council (http://www.msc.org/) as sustainable and well managed. For 40 years the effective management of this fishery has been aided by strong positive associations between the Southern Oscillation Index (SOI), strength of the Leeuwin Current, return of juveniles and abundance of the adult population [5], [6]. The strength of the Leeuwin Current is influenced by the El Niño/La Niña Southern Oscillation (ENSO) [7], and is a major factor in regional primary production along the southwest coast of Australia [8]. However, mechanisms driving the correlation between oceanography and recruitment remain unknown. Due to a significant downturn in recruitment to the fishery, and the apparent failure of the correlation between Leeuwin Current strength and larval settlement [9], [10], there is now significant interest in the environmental factors influencing the planktonic stages of *P. cygnus*.

**Figure 1:**
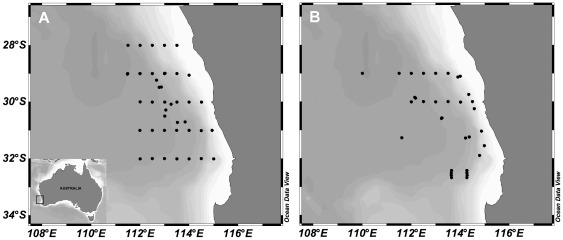
Study location. Sampling stations in the Eastern Indian Ocean occupied by the R. V. Southern Surveyor in A) July 2010 and B) August–September 2011, during oceanographic cruises designed to study the feeding ecology of phyllosoma larvae of the Western Rocklobster *Panulirus cygnus*.

The planktotrophic phyllosoma of *P. cygnus* have a pelagic larval duration of 9–11 months, during which time they metamorphose through 15 instars comprising 9 developmental stages [11]. Phyllosoma actively feed [12], amassing enough energy reserves to metamorphose to the non-feeding puerulus stage and then survive a weeklong migration to the coast where they settle as juveniles in benthic habitats [13]. The quality and abundance of food available to phyllosoma could therefore influence the numbers of larvae surviving the planktotrophic larval period [14] and metamorphosing to non-feeding pueruli, and the condition of pueruli settling at the coast [15], [16]. Settlement of pueruli is strongly linked to recruitment to the fishery 3–4 years later [6], [17].

The natural diet of spiny lobster phyllosoma has been the subject of significant debate (reviewed in [18], [19]) and has posed a challenge to researchers due to the cryptic morphology, behaviour, and relatively low abundance of phyllosoma in the pelagic environment [18]. Early oceanographic cruises reported that late stage *P. cygnus* phyllosoma larvae that were held in onboard aquaria fed on euphausiids [20]. However, quantitative studies at sea have not previously been reported due to the significant logistic issues surrounding experimentation at sea, including successful capture of patchy, rare and delicate larvae and live natural prey, adequate aquarium facilities, prohibitive costs and limited time associated with ship-based oceanographic research.

Studies of feeding behavior have typically been conducted in aquaculture facilities, or inferences of feeding made through indirect means from specimens sampled at sea. For example, fatty acid profiles [15], [21], [22], [23], nitrogen isotope composition [24], and molecular techniques [25], [26] have been used on various rock-lobster species to suggest that phyllosoma are opportunistic feeders and that soft prey including cnidarian jellies and salps may be important contributors to the diet. Similarly, studies of mouthpart and gut morphology of phyllosoma have suggested that the larvae might be better suited for feeding on soft foods, such as salps [18], [27]. Experimentally reared larvae consume a wide variety of prey items [28], [29], [30], and in aquaculture the phyllosoma of many Rocklobster species are typically fed on *Artemia* spp. in combination with mussel gonad [31]. In culture, the survival through to late stages of *P. cygnus* has typically been poor, generating interest in identifying the nutritional requirements and natural prey of phyllosoma. Despite the fact that the Western Rocklobster represent 20% of the value of Australian wild fisheries [4], there has been relatively little research on the ecology of *P. cygnus* larvae compared to other spiny lobster species, such as *Panulirus ornatus*, and their wild prey preference remains unknown.

The overall objective of this study was to identify wild prey of mid-late-stage phyllosoma of the Western Rocklobster *P. cygnus*. Shipboard feeding experiments were conducted during two survey programs designed to sample linkages between oceanographic processes, food webs, and distribution and abundance of *P. cygnus* larvae in the eastern Indian Ocean, off the coast of Western Australia (110°–155°E) between 28.0–32.5°S. Rates of consumption of selected prey items by mid-late stage *P. cygnus* phyllosoma were quantified in aquaria under various conditions (single prey type, choice of prey, and varying concentration of prey). Using this experimental framework two hypotheses were tested: 1) that phyllosoma demonstrate prey preference through variations in rate of prey consumption; and 2) that phyllosoma fed their “preferred” prey improved condition relative to phyllosoma fed other types of prey and to starved control. Condition was estimated by measuring the total lipid and fatty acid content of phyllosoma. This study provides the first evidence that phyllosoma of *P. cygnus* demonstrate marked prey preference, and that when fed this prey there are changes in their fatty acid profile and a tendency to accumulate greater amounts of lipid – results that could have implications for fisheries management and aquaculture of this commercially important species.

## Materials and Methods

### Experimental conditions and specimen collection

Sampling and experiments were conducted onboard the Australian National Marine Facility R.V. Southern Surveyor from 6–27 July 2010 (SS2010-v05) and 25 August–13 September 2011 (SS2011-v04), during surveys designed to sample the distributions, abundances, and trophic dynamics of *P. cygnus* phyllosoma and their prey field. The ship occupied 47 and 36 oceanographic stations in 2010 and 2011, respectively, from the coast (115°E) westwards to 110°E, and between 28.0–32.5°S (Fig. 1). Sea surface temperature during the cruises ranged from 17–22°C, and sea surface salinity was 34 to 36 PSU. Specimen collection was conducted under Murdoch University permit number R2338/10 and experiments were conducted under University of Western Australia ethics approval # AEC – RA/3/100/969.

*Panulirus cygnus* phyllosoma and prey for feeding experiments (see below) were collected at night (typically from 21:00–03:00) to maximize capture rates of phyllosoma [32], using a surface net with 1 m^2^ opening, 1 mm mesh, and 355 µm mesh hard cod end. The net was towed at 0–5 m depth at approximately 1 m s^−1^ for a nominal duration of 15 minutes. Upon retrieval of the net, the cod end was quickly removed and contents transferred into sorting trays. Mid-late stage *P. cygnus* phyllosoma were identified according to Brain et al. [11], and immediately placed into 15-L round aquaria with flow-through seawater at ambient temperatures without food, where they were monitored for 24–48 hours prior to inclusion in feeding experiments. In 2010, flow-through seawater was obtained from ∼5 m depth through the ship's firehose intake, and passed through a 5 µm inline cartridge filter (XStream Water). In 2011, a change in the ship's intake hose system necessitated inclusion of an inline degassing system to prevent gas supersaturation of the seawater. Feeding experiments were conducted in 4-L kreisel aquaria made of clear plexiglass with flow-through water, with 100% seawater exchange every 15 minutes.

Three potential prey types were selected for use in experiments: krill (primarily *Euphausia recurva, E. mutica*, and *E. gibba*), salps (primarily *Ihlea magalhanica*), and chaetognaths (primarily *Flaccisagitta enflata*) (15.4±3.8 mm; 11.4±3.1 mm; 20.3±3.0 mm, mean body length ± SD for each prey type, respectively, n = 50). These three potential prey types were selected based on: 1) published reports of potential prey of larvae of lobster species [15], [18], [19], [20], [21], [22], [23], [24], [25], [26], [27], [29], [30], [33]; 2) a pilot study where phyllosoma were presented various planktonic items and their behavioral response observed; and 3) the relative availability of similarly sized prey in plankton tows during the survey (potential prey within the appropriate size ranges abundant at all sampling locations). The longer body length of chaetognaths compared to krill or salps was off-set by their narrow morphology, such that body volume of the three prey species was similar.

**Figure 2:**
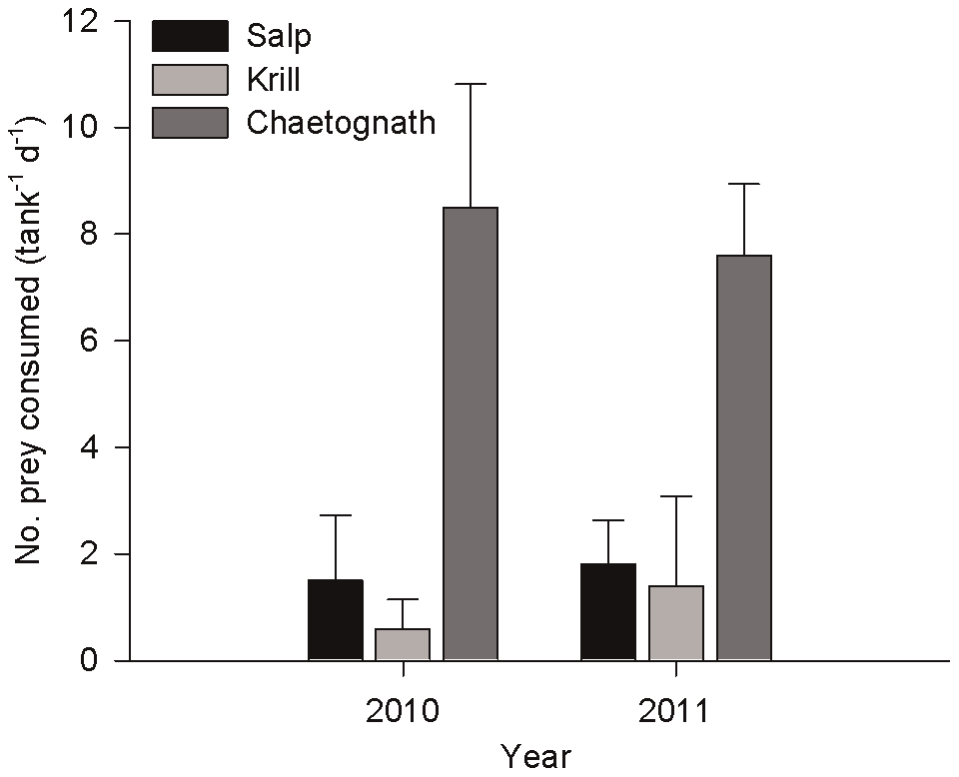
Consumption rate of 3 species of prey by *Panulirus cygnus* larvae in Prey Choice experiments. Consumption rate of 3 prey types (salps, krill, chaetognaths) by mid-late-stage phyllosoma of the Western Rocklobster *Panulirus cygnus* in ship-board feeding trials conducted in the eastern Indian Ocean in July 2010 and September 2011. In the prey choice experiment, 10 individuals of each of the 3 prey species were included in 4-L aquaria containing either 4 (2010) or 3 (2011) phyllosoma fed over 24 hours. n = 5 aquaria, mean+SD.

In all experiments (see below), a known number of individuals of live potential prey were added to aquaria within 15 minutes of collection of the prey items. Phyllosoma and prey were maintained in aquaria for a period of 24 hrs, at which point the unconsumed prey were enumerated. Prey was quantified as “consumed” if individuals were absent or if there were clear signs of predation (e.g., for krill, obvious bite marks on the soft tissue). After every 24 hr period unconsumed prey and any dead phyllosoma were removed from aquaria.

#### Prey choice experiment

Consumption rate of the prey species was quantified when all three species were available to the phyllosoma. In both 2010 and 2011, thirty individual prey items, comprised of 10 individuals from each of the 3 prey species, were provided to phyllosoma in each of 5 replicate aquaria (n = 4 or 3 phyllosoma in 2010 and 2011, respectively). In addition to the number of prey remaining after each 24 hr period, the prey species first captured by a phyllosoma, defined as the observation of prey being held in the maxilla, was observed for 60 minutes immediately following the addition of new prey.

To assess preference by phyllosoma for a particular prey, the Ivlev electivity index (*E*) [34] was calculated for each tank:
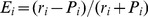
(1)where *r_i_* is the relative abundance of prey (*i*) in the diet and *P_i_* is the prey's relative abundance in the environment (tank). Differences in electivity of prey consumed in each year (2010, 2011) were tested using Generalized Least Squares (GLS), with electivity as a fixed factor (3 levels), and a term to account for heterogeneous variance as detected by examination of diagnostic plots. Homogeneous subsets were identified by visual inspection of means +/− S.E. of coefficients.

#### Single prey experiment

In 2010, experiments were set up to compare the consumption rate of each of the three potential prey by phyllosoma; 10 individuals of each prey type were introduced into 5 replicate aquaria per prey (15 tanks total) each containing 4 phyllosoma predators over 6 consecutive 24 hr periods. After the 6 day study period the mid-late stage phyllosoma used in these single prey feeding experiments were placed in individual glass vials and frozen at −20°C for later analysis of the fatty acid content (see below). As a control, phyllosoma freshly captured (T = 0), and phyllosoma maintained in aquaria for 6 days in the absence of food (control) were also analysed for fatty acid content. In addition, to characterize the fatty acid content of potential prey, aggregate samples containing 3–5 individual salps, krill or chaetognaths (n = 2–3 samples per prey species) were analyzed.

The effect of time and prey type on proportion of prey consumed was tested using a generalized linear model assuming a binomial distribution and using a logit-link function. Prey species, day and tank were included as a categorical fixed factor (3 levels), continuous fixed factor, and random factor, respectively. This approach was used to account for reductions in the number of phyllosoma in particular tanks through time due to mortality. The number of phyllosoma surviving in each tank on each day was used to offset the modeled proportion consumed. The model was fitted using the Laplace approximation with the lme4 package in R.

#### Prey encounter experiment

Given that *P. cygnus* phyllosoma are encounter feeders and both prey size and prey behavior will affect prey consumption rates, in 2011 the encounter rate (without predation) with each of the 3 prey species was quantified when all three species were available to the phyllosoma. Fifteen individual prey items, comprised of 5 individuals from each of the 3 prey species, were provided to 4 phyllosoma in each of 4 aquaria. A prey–phyllosoma encounter was defined as contact by prey with the ventral surface of a phyllosoma. When an encounter occurred, prey items were gently removed from the ventral surface of each phyllosoma to prevent consumption. The prey-phyllosoma encounters were counted for 10 minutes. Differences between the expected and observed number of encounters between phyllosoma and prey were tested using a Chi-square test.

#### Prey concentration experiment

To examine the effect of prey abundance on consumption rate, single prey diets consisting of 1, 5, 10, 20 and 30 chaetognaths per aquarium were provided to phyllosoma (4 phyllosoma per aquaria; 3 aquaria per prey concentration treatment, 5 treatments, for a total of 15 aquaria) and the number consumed per aquaria in a 24-hr period was quantified. The effect of concentration of prey on the proportion of prey consumed was tested using a generalized linear model assuming a binomial distribution and using a logit-link function.

**Figure 3:**
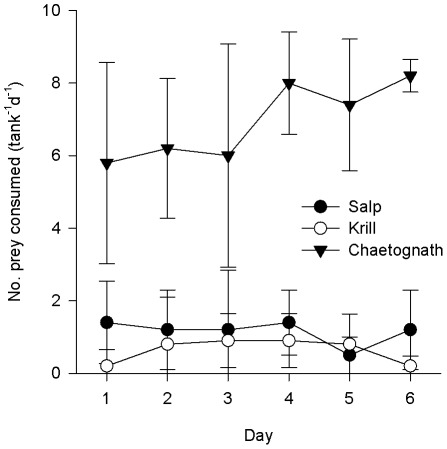
Consumption rate of 3 prey types by *Panulirus cygnus* larvae in Single Prey experiments. Consumption rate of 3 prey types (salps, krill, chaetognaths) by mid-late-stage phyllosoma of the Western Rocklobster *Panulirus cygnus* in ship-board feeding trials in the eastern Indian Ocean in July 2010, in *Single prey* experiments (10 individual prey per 4 L aquaria containing 2–4 phyllosoma, remaining prey removed and replaced every day for 6 days). n = 5 aquaria, mean+SD.

#### Characterization of prey field

Plankton samples for surveying the prey field in 2010 were taken with a depth stratified opening and closing EZ net, fitted with 10 nets of 335 µm mesh (mouth area 1.0 m^2^). A flowmeter positioned in front of the net was used to calculate the volume of seawater sampled by each net during each tow. The net was towed at a ship speed of ∼1 m s^−1^ during both day and night. Plankton were collected from 4 depths (1–50, 50–100, 100–150, and 150–200 m) at 5–7 stations along each transect. Plankton samples for the prey field survey were fixed in 10% buffered formaldehyde, and later sorted with the aid of a dissecting microscope. Quantitative counts of potential prey items for *P. cygnus* phyllosoma (see above) were determined on whole sample aliquots. Where samples contained a very large number of prey items, they were split using a Folsom splitter, and a fraction of the samples were counted. The counts of prey items were standardised to the volume of seawater sampled, and for the purposes of this study are reported as individuals m^−3^ (mean +/− SD) for each transect with the data pooled across depths and stations.

The effect of latitude on the abundance of prey was tested using the non-parametric Kruskal-Wallis test, with homogeneous subsets tested using the Mann-Whitney *U*-test. Differences in the abundance of chaetognaths compared to krill and salps (2010 data, pooled among transects) were tested using non-parametric Mann-Whitney *U*-tests.

#### Fatty acid laboratory analysis

Phyllosoma samples were defrosted, removed from the glass storage jars, blotted on tissue paper and then weighed. They were then extracted quantitatively by sonicating (3×10 mL) in glass tubes with a modified one-phase DCM-MeOH-Milli Q water Bligh and Dyer solvent mixture [35]. The extracts were transferred to a separating funnel (left for 4 hours) and after phase separation, the lipids were recovered in the lower DCM layer (solvents were removed in vacuo) and were made up to a known volume and stored, sealed in vials under nitrogen at −20°C.

The total neutral fraction containing sterols, phytol, *n*-alkanols and other compounds were obtained by alkaline saponification of an aliquot of the total lipids. Sterols, phytol and alcohols were converted to their corresponding O-TMSi ethers by treatment with *bis*(trimethylsilyl)trifluoroacetamide (BSTFA, 100 µL, 60°C, 60 min) and then stored in vials at −20°C.

**Figure 4:**
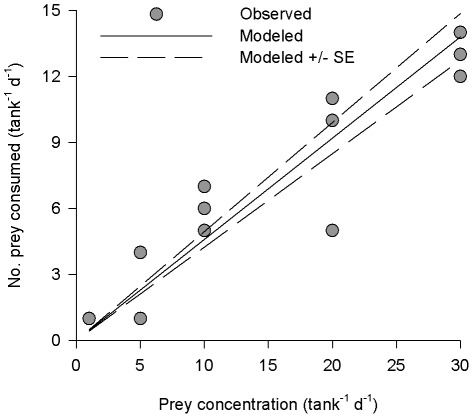
Effect of prey concentration on consumption by *Panulirus cygnus* larvae. Consumption rate of chaetognaths by mid-late stage phyllosoma of *Panulirus cygnus* as a function of prey concentration. Wild caught phyllosoma (4 individuals per 4 L aquarium) were fed varying concentrations of chaetognaths in ship-board feeding trials in the eastern Indian Ocean off the coast of Western Australia during a research cruise in July 2010. Data points indicate results from individual tanks (n = 15; some points overlapping). Black lines indicate the modeled relationship between proportion of prey consumed and concentration of prey (+/− S.E.).

The fatty acid fraction was treated with MeOH∶HCl∶DCM (10∶1∶1 v/v/v) at 80°C for 2 hours and the resulting Fatty Acid Methyl Esters (FAME) were extracted into hexane: DCM (4∶1, v/v). This fraction was also stored sealed in vials under nitrogen at −20°C ready for analysis using gas chromatography. Capillary Gas Chromatographic (GC) analyses were undertaken using a Varian 3800 GC fitted with a Flame Ionisation Detector (FID). 5α(H)-Cholestan-24-ol and 23:0 FAME were used as internal standards for quantification of the neutral lipids and fatty acid methyl esters respectively. The GC was equipped with a 50 m×0.32 mm i.d.×0.17 µm film thickness cross-linked 5% phenyl-methyl silicone (HP5 Ultra2) fused-silica capillary column. Helium was used as the carrier gas. Peak identifications were based on comparison of retention time data with data obtained for authentic and laboratory standards. Peak areas were quantified using Varian Galaxie chromatography software.

The identity of individual compounds was confirmed by gas chromatographic-mass spectrometric analysis (GC-MS) on a Thermoquest/Finnigan DSQ Trace benchtop mass spectrometer fitted with a direct capillary inlet and an on-column injector. Data were acquired in scan acquisition or selective ion monitoring and processed using Xcalibur software supplied with the instrument. The nonpolar column (HP5 Ultra) and operating conditions were similar to that described above for GC-FID analyses.

The effect of prey type (5 levels) on mean proportion of total lipids and individual fatty acids present in individual phyllosoma were examined using one-way ANOVA with prey-type as a fixed factor. Treatment levels included larvae freshly wild caught larvae (T = 0); larvae fed chaetognaths, krill or salps; and starved controls. The effect of prey species on particular fatty acids was tested using one-way ANOVA with prey species (3 levels) as a fixed factor. Where significant differences among treatments were identified, differences between pairs of treatment means were resolved using Tukey's test.

Statistical analyses were conducted using R 2.13.1 and SPSS 19.

## Results

### Prey consumption rate

When presented with a choice of prey in mixed prey diets, phyllosoma consumed more chaetognaths than either salps or krill [Electivity Index (mean ± SD) and results from GLS analysis. 2010: Chaetognaths = 0.40±0.09; Krill = −0.74±0.24; Salps = −0.47±0.40; t = 9.6, p<0.001; 2011: Chaetognaths = 0.36±0.08; Krill = −0.59±0.42; Salps = −0.35±0.20; t = 10.56, p<0.001] (Fig. 2). In 2010, the phyllosoma in the experiments were observed for the first 60 minutes of the 24 hr trial, and in all cases (n = 20 phyllosoma) the first species observed to be preyed upon was a chaetognath. The first predation occurred within 5 seconds to 45 minutes of provision of prey. No salps and krill were observed to be preyed upon within the first 60 min observation period, however, they were occasionally observed being held by pereiopods of phyllosoma over the following 23 hrs.

Similarly to the prey choice experiments, when phyllosoma were presented with single prey types there was higher consumption of chaetognaths compared to both krill and salps (for each p<0.001) (Figure 3). The proportion of prey consumed increased with time (p = 0.002), a result likely driven by an increase in the consumption rate of chaetognaths, but not of the other prey, over the week of experimentation (Figure 3).

Phyllosoma were observed to encounter chaetognaths more frequently than the other prey species [4.5±1.3 and 1.8±0.3 times (mean ± SD) more frequently than krill and salps, respectively]. Out of 207 observed encounters, there was a significant difference between the observed number of encounters between phyllosoma and the 3 prey species compared to a null model of equal probability of encounter (*X* ^2^ = 27.0, df = 2, p<0.001), with phyllosoma encountering chaetognaths about 1.6 times more frequently than expected.

There was a linear increase in the number of prey consumed over a 24 hr period with increasing concentration of prey (Figure 4). There was no indication that the relationship saturated with single prey diets consisting of 1, 5, 10, 20 and 30 chaetognaths per aquarium. There was no effect of the concentration of prey on the proportion of prey consumed (df = 14, p = 0.32). Phyllosoma consumed approximately 46% of available chaetognaths (slope of the GLM 0.46±0.04) regardless of concentration, indicating that proportional consumption of prey in the aquaria was independent of the concentration of prey, and that consumption increased with encounter probability.

### Prey abundance

**Figure 5:**
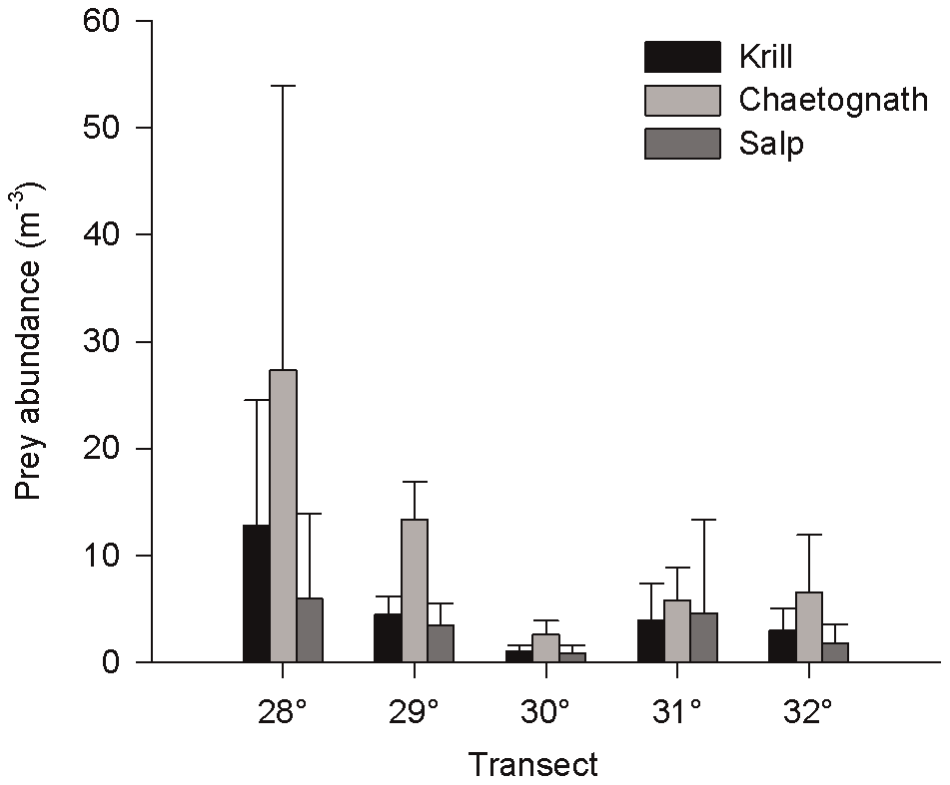
Distribution and concentration of planktonic prey of *Panulirus cygnus* larvae. Concentration of potential prey of Western Rocklobster phyllosoma in the Eastern Indian Ocean during a cruise conducted in July 2010. Samples were collected in depth-stratified tows between 0–200 m depth, using an opening-closing EZ net with 335 micron mesh size. Bars indicate mean ± SD counts per m^−3^ from depth stratified tows (pooled) at 5–7 stations along each transect.

The concentration of potential wild prey items in 2010 (krill, chaetognaths and salps) pooled from all depths and transects ranged from 0.8 to 27.3 prey m^−3^ (Figure 5). The abundance of krill, chaetognaths and salps varied with latitude (p<0.001), with highest prey abundance on the northern transects (latitude 28 and 29°S). Overall, throughout the 2010 survey, abundance of chaetognaths was variable, but consistently outnumbered abundance of krill and salps (*U* = 256, p = 0.004 and *U* = 164, p<0.001, respectively). The abundance of other soft-bodied potential prey items, such as siphonophores (1.2–4.4 m^−3^), heteropods (0.002–0.012 m^−3^), medusa (0.005–0.025 m^−3^), squid larvae (0.01–0.06 m^−3^) and fish larvae (0.2–2.2 m^−3^) were generally recorded at much lower abundances. Including all the potential prey items that we enumerated, chaetognaths comprised up to 49% of the potential prey field.

### Fatty acid composition

**Figure 6:**
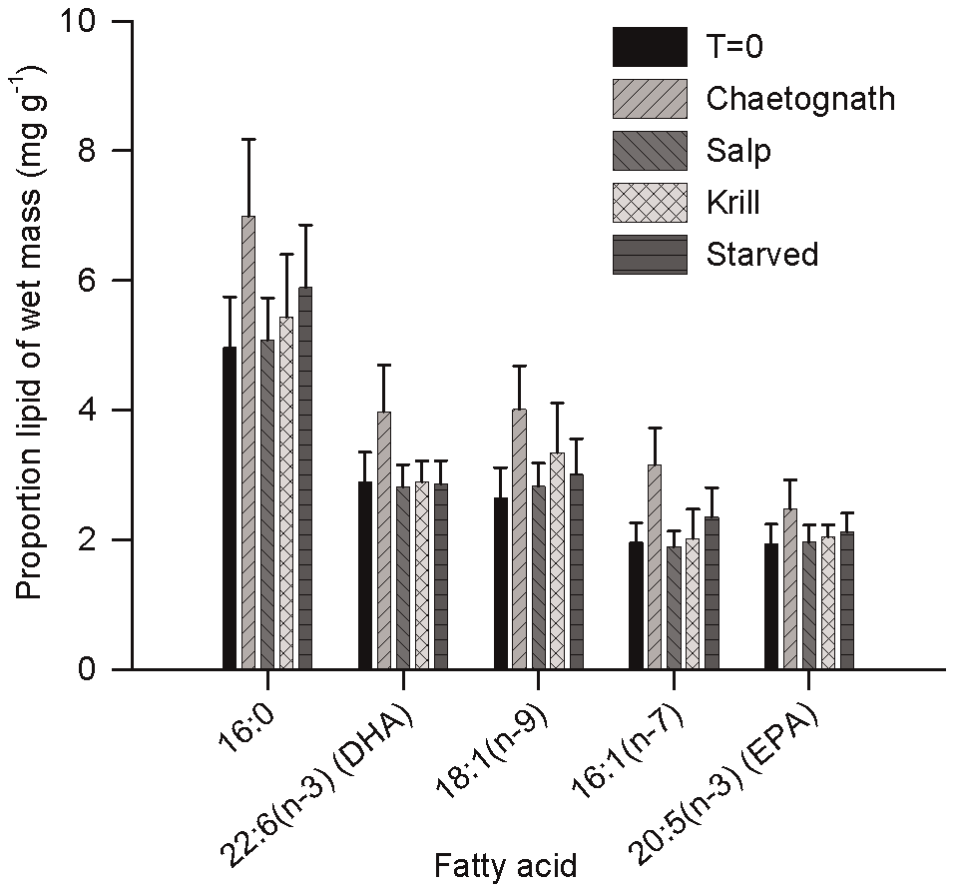
Proportional abundance of dominant fatty acids in *Panulirus cygnus* larvae fed various diets. Proportional abundance (mean ± SE) of the five most dominant fatty acids present in *P. cygnus* phyllosoma used in ship-board feeding trials in the eastern Indian Ocean in July 2010. There was a non-statistically significant trend towards higher proportion lipid of wet mass for each particular fatty acid in phyllosoma fed chaetognaths, compared to phyllosoma fed salps or krill, freshly caught (T = 0), or starved (control) [16:0, F_(4,30)_ = 0.84, p = 0.51; 22:6(n-3) (DHA), F_(4,30)_ = 1.12, p = 0.37; 18:1(n-9), F_(4,30)_ = 1.05, p = 0.40; 16:1(n-7), F_(4,30)_ = 1.60, p = 0.20; 20:5(n-3) (EPA), F_(4,30)_ = 0.47, p = 0.76].

Freshly caught mid-late stage phyllosoma displayed large variability in their lipid content and fatty acid composition. For freshly caught phyllosoma (T = 0) the total lipid content of phyllosoma ranged from 8.0 to 45.0 mg g^−1^ of wet mass. The dominant fatty acids among the T = 0 sample were 16:0, 22:6(n-3) (DHA), 18:1(n-9), 16:1(n-7), 20:5(n-3) (EPA) with means of 4.96±0.78 S.E., 2.90±0.45, 2.65±0.47, 1.96±0.30 and 1.94±0.30 mg g^−1^ of wet mass, respectively. The mid-late stage phyllosoma held in aquaria and fed single species diets for 6 days (T = 6) showed no significant changes in their total lipid content relative to both T = 0 and starved controls (F_4,30_ = 0.90; p<0.48). However, the phyllosomas experimentally fed chaetognaths tended towards higher mean total lipid (T = 0: 20.4±3.0 S.E. mg g^−1^; Starved: 23.1±3.4; Krill: 21.8±3.0; Salp: 20.5±2.3; Chaetognath: 28.6±4.6) and most individual fatty acids as a proportion of their wet mass compared to all other feeding treatments, including starved controls (Figure 6), although the results were not statistically significant. ANOVA could detect significant differences among feeding treatments for the proportion of some minor fatty acids present in phyllosoma, with means for i16:00 (F_4,30_ = 4.62; p<0.005), 15:01 (F_4,30_ = 3.17; p<0.03), 16:04 (F_4,30_ = 5.33; p<0.005), C24:1 (F_4,30_ = 6.89; p<0.0005) significantly elevated, and the mean for 16:03 significantly (F_4,30_ = 4.33; p<0.007) depressed in the chaetognath feeding treatment. These changes in the phyllosoma that had been fed chaetognaths reflected the relative mean abundances of these specific minor fatty acids that were present within the sample of analysed chaetognaths.

After 6 days the control starved phyllosoma showed no significant differences in mean total lipid content, lipid saturation groups (poly-, mono- and unsaturates) or individual fatty acids compared to phyllosoma at T = 0 (for each test p>0.05) (Figure 6).

Prey had markedly different overall lipid content with krill having significantly more lipid than both chaetognaths and salps (F_(2,7)_ = 22.1, p<0.003, 7.19±0.64, 3.46±0.70, 1.53±0.33 mean ± S.E., lipid mg g^−1^ wet mass, for krill, chaetognaths and salps respectively). Overall, the fatty acid profiles of these three potential prey species were broadly similar, although chaetognaths tended to have a greater proportion of their lipid as monosaturated fatty acids (especially 18:1(n-9) and 16:1(n-7)), than krill and salps, which tended to have a greater proportion of their lipid as polyunsaturated fatty acids (especially DHA – 22:6(n-3) and EPA – 20:5(n-3)).

## Discussion

This study provides the first direct documentation of preferential feeding behaviour of wild caught phyllosoma of the Western Rocklobster *P. cygnus*. In ship-board feeding trials mid-late-stage phyllosoma of *P. cygnus* consumed more chaetognaths than two other types of abundant potential prey items, both in single prey as well as in prey choice experiments. Chaetognaths were the first prey consumed when provided a selection of different prey items, and the time until first capture of chaetognaths ranged from seconds to 45 min. In contrast, krill and salps were consumed in low numbers within 24 hours, and they were never consumed within the first 1 h of experimental observation. Although chaetognaths were more likely than salps and krill to be encountered by phyllosoma in the encounter rate experiment (1.8–4.5 times more frequently than the other prey, respectively), it was insufficient to explain the marked feeding preference (2–8 times more) for chaetognaths demonstrated by the phyllosoma.

Although consumption of chaetognaths by other species of Palinuridae and Scyllaridae lobsters has previously been observed in laboratory settings [28], [36], [37], such a pronounced preference by *P. cygnus* phyllosoma for a particular prey has not previously been documented. Mitchell [28] conducted laboratory feeding trials by offering wild plankton to larvae of the California spiny lobster *Panulirus interruptus*, which demonstrated preference for medusae, ctenophores, chaetognaths, and other soft-bodied food. In laboratory studies, diets of chaetognaths provided adequate nutrition for instar 5 of the Japanese spiny lobster, *Panulirus japonicus* [36], and for instar 1 of the California spiny lobster *Panulirus interruptus* [37]. In contrast, Suzuki and colleagues [25] examined DNA in the hepatopancreas of wild caught *Panulirus japonicus* phyllosoma in the Ryukyu Archipelago, Japan, but were surprised to not find evidence of consumption of chaetognaths. DNA from chaetognaths was subsequently detected in wild caught *P. japonicus* [38].

Inferences of prey consumption by *P. cygnus* phyllosoma have been previously made through indirect means such as fatty acid analyses (e.g. [15], [22]). Based on fatty acid analyses, Phleger et al. [21] suggested that a significant composition of the diet of phyllosoma of the Southern Rocklobster, *Jasus edwardsii*, was large gelatinous zooplankton, such as cnidarian jellies or salps. More recently, based on nitrogen isotope composition and fatty acid profiles of wild caught *P. cygnus* phyllosoma and pueruli, it was suggested that they consume grazers of diatoms produced in a ‘classic’ nitrate-based food chain [23], [24]. However, work by Phillips et al. [15] suggested that diatoms were relatively unimportant in the phyllosoma diet, and that the microbial food web and gelatinous zooplankton such as salps were more likely to be important food sources. Jeffs et al. [22] used fatty acid profiles to identify the mixed prey of *Jasus edwardsii*, suggesting opportunistic feeding; however, they also suggested that the use of fatty acids and sterols to identify prey of phyllosoma had major limitations. Suzuki et al. [26] and Chow et al. [38] used molecular techniques to examine the gut contents of the phyllosoma of several palinurid and scyllarid lobster species and commonly detected DNA material from cnidarians and urochordates, although a signal matching chaetognaths (*Sagitta* sp.) was also detected.

In the current study the rate of consumption of chaetognaths increased with prey concentration, a finding that is consistent with studies using *Artemia* as prey [39]. Plankton, including *P. cygnus* phyllosoma and their potential prey, are distributed unevenly in the ocean and their patchiness is driven by both biological and physical processes. The concentration of chaetognaths quantified in the field survey was similar to those in the same region in 1992–1993 [40]. Chaetognaths comprised a relatively large fraction, up to 49%, of the macro-zooplankton sampled during this cruise, suggesting that they are a non-trivial component of the pelagic food web. The abundance of chaetognaths varies seasonally [41], with highest abundance occurring in the autumn/winter, the season during which this study occurred. The concentrations of prey provided in the feeding study were 2–3 orders of magnitude higher than those occurring naturally in the field (0.25–7.5 L^−1^, vs. 0.003 L^−1^), yet consumption did not plateau with increasing prey concentration, suggesting that food could be limiting to *P. cygnus* phyllosoma in the wild. Thus, patchiness of prey distribution in the ocean could potentially influence condition of wild phyllosoma and their ultimate recruitment to the fishery.

There are several limitations to our study. Firstly, obtaining intact phyllosoma from the surface plankton net was difficult, and indeed some of the specimens used in this study lacked 2–6 pereiopods. However, phyllosoma successfully captured actively swimming chaetognaths preferentially over the more slowly moving salps, suggesting that pereiopod loss did not affect their choice of prey. Secondly, for logistical purposes, the number of individual prey was quantified rather than the mass or energetic content of prey consumed. Such measurements would be more amenable to laboratory studies than to ship-board feeding trials. Thirdly, it is likely that phyllosoma in the wild consume species that we did not include in this study, however, logistical constraints (insufficiently abundant prey, tank space) prevented the inclusion of additional prey types in these experiments. In a pilot study *P. cygnus* phyllosoma were presented with various other food types, including cnidarian jelly fish, ctenophores, copepods, pteropods, radiolarians, heteropods, cephalopods, and amphipods, and did not observe a clear feeding response similar to that of the response to chaetognaths. Lastly, feeding experiments in aquaria are influenced by artifacts inherent in this type of behavioural research, such as unrealistic current velocities, higher concentrations of prey, and the presence of walls; direct inferences to open-ocean feeding behavior should consider these experimental limitations.

The total lipid content and fatty acid composition of wild caught mid-late stage phyllosoma was highly variable, a common feature of the phyllosoma of a range of spiny lobster species, and is probably a reflection of their recent feeding history, especially due to the limited availability of lipid-rich prey in oligotrophic waters [15], [21], [22]. The profile of the dominant fatty acids in wild mid-late stage phyllosoma (i.e., 16:0, 22:6(n-3) (DHA), 18:1(n-9), 16:1(n-7), 20:5(n-3) (EPA)) were consistent with those previously identified for this species [15], and similar to those reported for *J. edwardsii* larvae and pueruli, and *Sagmariasus verreauxi* pueruli [21], [22], [42].

Commonly used diatom fatty acid indicator ratios [14:0+16:1+C16PUFA/16:0 = mean of 0.6%), [16:1(n–7)/16:0 = mean of 0.4%], (C16 FA/C18 FA = mean of 1.4%), (16:1/18:1 = mean of 0.7%), and (C16PUFA/C18PUFA = mean of 0.6%) were low to moderate in the mid-late stage larvae, indicating that diatoms were not a major contributor to their food chain [43], [44], [45]. The dinoflagellate markers [18:4(n–3) = mean of 1.2%, 18:1(n–9) = mean of 12.7%, 22:6(n–3) = mean of 14.1%] [45], [46] were also relatively low to medium. Both the general flagellate (autotrophs and heterotrophs) marker (C18 FA = mean of 26.3%) and the bacterial marker ([15:0+i15:0+a15:0+17:0+i17: 0+a17:0+18:1(n–7)] = mean of 5.6%) were high [43], [45], [47]. The general lack of the copepod signature 20:1(n–9)c (mean of 0%) and 22:1(n–11) (mean of 0%) would indicate that copepods are not a significant contributor to the food chain of *P. cygnus* larvae [15]. The absence of copepod biomarkers in chaetognaths, indicates that despite this taxon primarily being associated with copepod predation [48], that in these waters chaetognaths are potentially adopting a different role in the food chain in the absence of primary production to support sufficient copepod biomass. Overall, these results indicate that mid-late stage *P. cygnus* larvae are feeding in waters with low primary productivity and elevated bacterial production. Under such oligotrophic conditions, the greater reliance on microbial grazing becomes an important basis to the food web which is known as a ‘microbial loop’ [49].

When fed chaetognaths for one week, the phyllosoma tended to accumulate higher mean total lipid and most individual fatty acids as a proportion of their wet mass compared to phyllosoma starved or fed salps or krill. However, the high natural variability in the lipid composition of phyllosoma prevented any major differences in lipid composition of phyllosoma being detected among the experimental treatments. Significant differences were detected for the mean proportion of some minor fatty acids present in phyllosoma in the chaetognath feeding treatment (i.e., i16:00, 15:01, 16:04, 16:0, C24:1) which appeared to directly reflect specific and marked differences in the fatty acid profile of chaetognaths compared with krill and salps. The fatty acid profiles of phyllosoma are known to be strongly influenced by differences in their dietary intake. This has been confirmed experimentally by feeding cultured phyllosoma with brine shrimp (*Artemia*) enriched with different fatty acid profiles [50], [51], [52], [53]. Phyllosoma larvae are also known to have a high requirement for dietary sources of 22:6(n-3) DHA [21], [53], [54], [55], for which chaetognaths are clearly a rich source for *P. cygnus* phyllosoma (i.e., on average 21.9% of fatty acids in chaetognaths). Phyllosoma starved for 6 days showed little sign of change in their lipid content. This may be due to phyllosoma mostly using protein to meet their metabolic needs and conserving lipid, especially during periods of starvation [56], [57]. This may be a strategy by mid-late stage phyllosoma to protect valuable accumulated lipid energy stores which are known to be required to fuel the subsequent non-feeding puerulus stage [15], [23], [58].

The three prey items provided to phyllosoma experimentally had significantly different potential lipid yields, with krill offering about double the total lipid content of chaetognaths and salps for the same amount of wet mass consumed, on average 7.19 mg g^−1^ versus 3.46 mg g^−1^ and 1.53 mg g^−1^ respectively. In addition, krill tended to contain a higher proportion of polyunsaturated fatty acids especially, DHA and EPA, which are both important fatty acids for phyllosoma [23]. While krill may offer higher lipid nutritional reward as prey for phyllosoma, they may not be targeted because they are more difficult to consume because of the need to remove the carapace from the krill [18]. Therefore, any differences in potential lipid yield among the prey types would be compensated by the increased overall consumption of greater total wet mass of chaetognaths.

The results of this study contribute towards understanding the natural diet and nutrition of this commercially valuable species during its planktonic phase, and will provide a basis for future studies of Rocklobster feeding behaviour, population dynamics, fisheries, and aquaculture. Future studies should include: 1) identifying oceanographic mechanisms driving prey distribution and abundance; 2) examining sensory mechanisms influencing phyllosoma response to various prey types; 3) investigating nutritional characteristics of prey to inform aquaculture. Increasing consumption of prey with increasing concentration and rate of encounter suggests that phyllosoma are prey limited in the oligotrophic ocean, and that variations in ocean production could have a relatively strong impact on this commercially important species. Given the recent decoupling between the predictive model of sea level height in Fremantle and puerulus settlement, a mechanistic examination of the oceanographic features that contribute to phyllosoma success, including production and food availability, is clearly warranted.
